# Differential Evolution Optimization of Microwave Focused Hyperthermia Phased Array Excitation for Targeted Breast Cancer Heating

**DOI:** 10.3390/s23083799

**Published:** 2023-04-07

**Authors:** Cheng Lyu, Wenxing Li, Bin Yang

**Affiliations:** 1 College of Information and Communication Engineering, Harbin Engineering University, Harbin 150000, China; 2 School of Cyberspace, Hangzhou Dianzi University, Hangzhou 310018, China; yb_alonline@126.com

**Keywords:** breast cancer, differential evolution (DE), hyperthermia treatment planning (HTP), phased array excitation, specific absorption rate (SAR)

## Abstract

Microwave hyperthermia using the phased array applicator is a non-invasive treatment modality for breast cancer. Hyperthermia treatment planning (HTP) is critical to accurately treating breast cancer and avoiding damage to the patient’s healthy tissue. A global optimization algorithm, differential evolution (DE) algorithm, has been applied to optimize HTP for breast cancer and its ability to improve the treatment effect was proved by electromagnetic (EM) and thermal simulation results. DE algorithm is compared to time reversal (TR) technology, particle swarm optimization (PSO) algorithm, and genetic algorithm (GA) in HTP for breast cancer in terms of convergence rate and treatment results, such as treatment indicators and temperature parameters. The current approaches in breast cancer microwave hyperthermia still have the problem of hotspots in healthy tissue. DE enhances focused microwave energy absorption into the tumor and reduces the relative energy of healthy tissue during hyperthermia treatment. By comparing the treatment results of each objective function used in DE, the DE algorithm with hotspot to target quotient (HTQ) as the objective function has outstanding performance in HTP for breast cancer, which can increase the focused microwave energy of the tumor and decrease the damage to healthy tissue.

## 1. Introduction

Breast cancer has become the most common cancer in the world that harm women’s health. According to the cancer statistics of China in 2022 [1], breast cancer accounts for 19.54% of the cancer incidence of Chinese women, ranking first in cancer incidence. Mastectomy or partial mastectomy combined with radiotherapy, chemotherapy, hormone therapy, and other medical techniques are the main treatment methods for breast cancer. Hyperthermia [2,3] has attracted much attention in the treatment of breast cancer, prostate cancer, liver cancer, skin cancer, etc. Considerable attention has been paid to several thermal techniques (extreme cold or heat), including cryoablation [4], radiofrequency ablation (RFA) [5], interstitial laser therapy (ILT) [6], and high-intensity focused ultrasound (HIFU) [7], which apply thermal ablation to eliminate a malignant tumor and its margins. Ongoing phase II clinical trials are evaluating the efficacy of cryoablation for low-risk breast cancer (Luminal A) as an alternative to breast surgery; however, the drawback is that there is limited control (>1.5 cm^3^) over the size of the ablation zone near the metal probe. ILT and RFA are minimally invasive percutaneous technologies that destroy preclinical tumors through intense heat generated by focusing (>55 °C). In addition, RFA electrodes remains an ongoing challenge in penetrating hard fibrous tissue and controlling treatment thermal power. For larger breast tumors (>2 cm^3^), HIFU is challenging. Among the various thermal technologies, focused microwave hyperthermia therapy (FMHT) has emerged as a promising technique for breast cancer treatment. The advantages of FMHT are reduction of scarring, better preservation of healthy tissue, rapid postoperative recovery, and less medical costs. The results of clinical trials combining FMHT and radiotherapy demonstrated significantly improvement in the treatment of superficial breast cancer and chest wall recurrence.

The purpose of FMHT is to raise the temperature of the breast tumors over 42 °C for 60 min and to maintain healthy tissue at a safe temperature (<40 °C). In the last two decades, several phased array applicators have been reported for non-invasive microwave hyperthermia of breast cancer, operating in the ISM band (Industrial, Scientific, and Medical) [8] or other frequencies [9,10]. The ISM band includes 0.434 GHz [11], 0.915 GHz [8,12], and 2.45 GHz [13]. At present, there are few pieces of research on multi-resonance phased array applicators [14,15], while there are many designs of multi-band antennas [16,17] for FMHT of breast cancer. The multi-resonance phased array application adjusts the frequency of treatment according to the depth and volume of the tumor for better treatment results. The amplitudes and phases of treatment antennas in the phased array applicator were optimized using the hyperthermia treatment planning (HTP) process for targeting malignant tissues and ensuring the safety of healthy tissue.

HTP optimization methods optimize excitations of phased arrays based on SAR to optimize the energy distribution of tumor tissue and healthy tissue [18]. The common HTP optimization method is time reversal (TR) technology [8,11]. However, the energy loss in the propagation process of microwaves affects the optimization effect of TR, resulting in the shift of focus and unexpected hotspots in healthy tissues and other treatment problems. In recent years, global optimization algorithms are involved to optimize HTP for breast cancer, including the Nelder–Mead simplex (NMS) algorithm [12], pattern search (PS) [19], particle swarm optimization (PSO) [9,14], and genetic algorithm (GA) [20]. The particle swarm optimization (PSO) algorithm and differential evolution (DE) algorithm had been applied to optimize HTP for head and neck tumors [21]. The treatment indicators such as hotspot to target quotient (HTQ) [21,22], average power absorption ratio (aPA), and tumor coverage 50% (TC_50_) are used as the objective function of the optimization algorithm to improve tumor treatment outcomes. The mean SAR deposited in the tumor was also utilized as the objective function of the HTP optimization method in previous studies [22,23]. At present, single objective genetic algorithm (SOGA) and multi-objective genetic algorithm (MOGA), as HTP optimization algorithm, optimizes the excitations of an 18-element phased array applicator at 0.434 GHz for the treatment of large-sized breast tumors [20]. The results show that MOGA can reduce the excess hot spots of healthy tissue and the channel power in HTP. However, the convergence rate is slow and the focusing effect needs to be further improved.

The DE algorithm is proposed in this work, in order to improve the therapeutic effect of the dual resonant phased array applicator for breast cancer microwave hyperthermia at 0.915 GHz and 2.45 GHz. The phased array applicator is used to target 1 cm^3^ and 2 cm^3^ spherical breast tumors in the upper outer quadrant of general and heterogeneous breast models. Comparison using TR, PSO, and GA with DE to demonstrate the advantages of DE in HTP for breast cancer. The extraordinary optimization performance of DE was determined by evaluating the treatment results of four HTP optimization methods, including treatment indicators (HTQ, aPA, and TC_50_), SAR distributions, temperature parameters, and temperature distributions. Results show that compared with PSO and GA used for FMHT of breast cancer, DE provides better-focused treatment results, improves the treatment temperature of tumors, and reduces unexpected hotspots in healthy tissues. The effect of different SAR-related objective functions in DE on tumor therapy is analyzed in this paper. Finally, it is verified that the DE algorithm with HTQ as the objective function is the optimal HTP optimization algorithm for breast cancer.

The structure of this paper is shown as follows. The introduction of the HTP process, evaluation criteria of treatment effect, and the optimization algorithms employed in this paper are demonstrated in Section 2. The optimization results of DE, TR, PSO, and GA are reported in Section 3, as well as the optimization results of different objective functions used in DE. The discussion and the conclusion of this work are reported in Section 4 and Section 5, respectively.

## 2. Hyperthermia Treatment Planning

### 2.1. Process of HTP

The purpose of microwave hyperthermia is to selectively deposit microwave power in malignant tumors and to avoid extra power appearing in the healthy tissue. In our previous work, time reversal (TR) technology was utilized to optimize the excitation of the phased array. TR is affected by energy loss during the propagation of microwaves, and the therapeutic effect was not accurate, resulting in focusing deviation, excess hotspots in healthy tissues, and a large ablation range. Treatment options are acceptable if the tumor is completely thermal damaged and the thermal damage of surrounding healthy tissue is less than 5% [20]. Therefore, further optimization of treatment results is needed. The global optimization algorithms are used in HTP to reduce the energy of excess hotspots in healthy tissues and ensure a certain ablation range for improving the effectiveness of hyperthermia. Tissues in the breast have different dielectric properties depending on their water content, and tissues with higher water content can absorb more energy. Depending on the density of the breast, the contrast between the permittivity of the tumor and the surrounding tissue is also different. According to radiographic breast densities, the breast is classified into the extremely dense breast, heterogeneously dense breast, scattered fibro-glandular breast, and predominantly fatty breast. The therapeutic effect of phased array microwave hyperthermia for breast cancer is related to the contrast of dielectric properties between the tumor and surrounding tissue. In order to verify that the phased array optimized by the optimization algorithms has a good targeting ability, the tissue around the tumor is set as fiber glandular tissue. The tissue dielectric and thermal properties are shown in Table 1.

During the treatment, the energy density deposition in breast tissue is expressed as

(1) $$Q(r)=0.5\sigma (r){\left|\vec{E}(r)\right|}^{2}$$

In Equation (1), the $\vec{E}(r)$ is the total electric field of the breast tissue and σ(r) is the bulk conductivity of the tissue. The total electric field is given by

(2) $$\vec{E}(r)=\sum_{n=1}^{N}A_{n}e^{-i\varphi_{n}}{\vec{E}}_{n}(r)$$

where $A_{n}$ and $\varphi_{n}$ are the amplitude and phase delay of the *n*th antenna in the phased array, respectively. ${\vec{E}}_{n}(r)$ is the electric field provided by the *n*th antenna and *N* is the number of antennas in the phased array applicator (*N* = 36). The optimization algorithms are used to optimize the excitation of the phased array, so the EM distribution in the tissue is changed. The SAR can be expressed as

(3) $$\mathrm{SAR}=\frac{\sigma (r){\left|\vec{E}(r)\right|}^{2}}{2\rho (r)}=\frac{Q(r)}{\rho (r)}$$

where ρ(r) is the density of the tissue. According to Equation (3), the EM distribution can be known by analyzing the SAR value. Because of the nature of breast tissue included in the SAR calculation, the focus results can be observed through the SAR distribution. By selecting the treatment indicators calculated by SAR as the objective function of the optimization algorithms, the excess hotspot energy of healthy tissue is reduced and more energy is stored in the tumor, thus the purpose of optimizing the SAR distribution is achieved.

The breast model and the power density from the EM simulation are imported into COMSOL for thermal simulation. Because there are no large blood vessels in the breast, Pennes’ bioheat equation can be expressed as [24].

(4) $$\rho (r)C_{P}(r)\frac{\partial T(r,t)}{\partial t}=\nabla \left(k(r)\nabla T(r,t)\right)+\rho_{b}c_{b}\omega_{b}(T_{a}-T(r,t))+P(r)+Q_{m}$$

In Equation (4), *t* denotes time, $\rho_{b}$ is the density of blood, $c_{b}$ is the specific heat capacity of the blood, $\omega_{b}$ is blood perfusion rate, $C_{P}$ is the specific heat capacity, $Q_{m}$ is the tissue metabolic rate, and $T_{a}$ is the arterial blood temperature (37 °C) [25]. The thermal simulation was carried out for 60 min for the power density P(r), based on the duration of a typical hyperthermia treatment. The temperature equation includes the dielectric and thermal properties of the breast tissue, which is related to SAR. Hence, the results of treatment can be evaluated by temperature distribution and SAR distribution. Recent studies have found that heterogeneous blood perfusion during thermal treatment has an effect on temperature computation [26]. Due to the absence of large blood vessels in the mammary gland, the heterogeneous blood perfusion effects were not considered in our study [9].

### 2.2. Breast Model and the Phased Array Applicator

**Figure 1:**
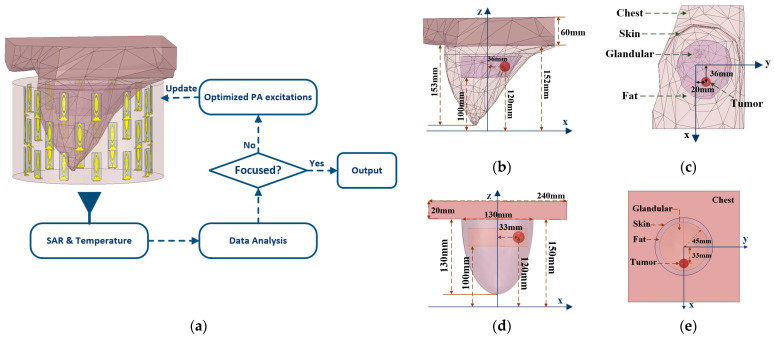
The process of optimizing dual-resonant phased array applicator in HTP and two breast models. (**a**) Flowchart of HTP employed in this work; (**b**) Side view of a heterogeneous breast model; (**c**) Top view of a heterogenous breast model; (**d**) Side view of a general breast model; (**e**) Top view of a general breast model.

The construction of the dual-resonant phased array applicator optimized by global optimization algorithms for breast cancer microwave hyperthermia is shown in Figure 1a. There are 12 × 3 ultra-wideband microstrip antennas uniformly arranged to form a cylindrical phased array. The diameter and height of the phased array are 200 mm and 150 mm, respectively. A cylindrical container with a diameter of 240 mm is filled with the coupling medium (oil-water mixture). The phased array applicator and the oil-in-water were placed inside the airbox. The surface of the air box was defined at a distance of λ/4 from the outer surface of the liquid. The perfectly matched layer boundary condition is adopted to absorb the outward-radiating electromagnetic wave incident on the surface of the air box boundary. The detail of the dual-resonant phased array applicator can be seen in our previous work [15]. The phased array applicator operates at two treatment frequencies (0.915 GHz and 2.45 GHz). By adjusting the frequency to change the heating range, the applicator can accurately treat 1 cm^3^ and 2 cm^3^ tumors. In this paper, DE, TR, PSO, and GA algorithms were used to optimize the phase and amplitude of the phased array applicator to achieve tumor targeting. The optimization is complete until the stop criterion or the maximum number of iterations is reached. The HTP optimization process is completed jointly by Matlab R2021a and HFSS. The SAR distribution obtained by EM simulation is returned to Matlab for objective function calculation to optimize the excitation of the phased array. The breast model and optimal power density were imported in COMSOL 5.3 for thermal simulation and the temperature distribution is obtained.

The general and heterogeneous breast models composed of adipose, skin, glandular, and tumor tissue were treated with a dual-resonant phased array applicator, as shown in Figure 1b–e. The heterogeneous breast model is extracted from the component library of Ansys Electronics 2019 (HFSS), as shown in Figure 1b,c. The general breast was modeled as a semi-ellipsoid with 1 mm thick skin and the chest wall side size is 20 mm of lateral dimensions as 240 × 240 mm^2^, as shown in Figure 1d,e. According to statistics, breast cancer is most likely to occur in the upper outer quadrant of the breast [27]. Therefore, 1 cm^3^ and 2 cm^3^ of the spherical tumors were located in the heterogeneous breast model at (36, 20, 120) and the general breast model at (33, 0, 120), respectively. Depending on the differences in the focusing range of different frequencies, 2 cm^3^ tumors were treated at 0.915 GHz and 1 cm^3^ tumors were heated at 2.45 GHz. The HTP optimization methods were used to optimize the excitation of the phased array applicator at two operating frequencies to improve the therapeutic effect of 1 cm^3^ and 2 cm^3^ tumors.

In the thermal simulation, the initial temperature of the breast was set as 37 °C and the skin surface was surrounded by a 20 °C coupling medium (oil-in-water) for keeping the safety of healthy tissue. A convective boundary condition was applied to contact the skin surface with the coupling solution, and the convective heat transfer coefficient was set as 250 W/K/m^2^ [20]. The temperature of the chest tissue exposed outside the applicator was also set to 37 °C. The steady-state temperature distribution was used to calculate the temperature parameters.

### 2.3. Treatment Indicators

The treatment indicators are proposed to determine the quality of treatment. SAR values are obtained in EM simulation for calculating hotspot to target quotient (HTQ), average power absorption ratio (aPA), and tumor coverage 50% (TC_50_), where they are defined as

(5) $$\mathrm{aPA}=\frac{\sum P_{\mathrm{tumor}}/V_{\mathrm{tumor}}}{\sum P_{\mathrm{healthy}}/V_{\mathrm{healthy}}}$$

where $P_{\mathrm{tumor}}$ and $P_{\mathrm{healthy}}$ present the power absorbed in the tumor and healthy tissue, respectively [11]. $V_{\mathrm{tumor}}$ and $V_{\mathrm{healthy}}$ are the volume of tumor and healthy tissue, respectively. The treatment indicator aPA represents the ratio of the average power of the tumor to the average power of healthy tissue. Equations (4) and (5) quantified relative power deposition in tumor target tissue and healthy tissue. A higher aPA value means a better hyperthermia result. HTQ is expressed as

(6) $$\mathrm{HTQ}=\frac{{\bar{\mathrm{SAR}}}_{V_{{}_{1}}}}{{\bar{\mathrm{SAR}}}_{\mathrm{target}}}$$

where V_1_ is the volume of the top 1% of healthy tissue with the highest SAR. HTQ is used to measure the ratio of the mean SAR value of V_1_ to the mean SAR value of tumor. The smaller HTQ, the better the therapeutic effect. Generally, HTQ is used as the objective function of the optimization algorithms in HTP to improve the power in the tumor and reduce the residual heat in the healthy tissue. TC_50_ is expressed as

(7) $${\mathrm{TC}}_{50}=\frac{V_{\mathrm{target}}\left(\mathrm{SAR}>\max(\mathrm{SAR})/2\right)}{V_{\mathrm{tumor}}}\%$$

TC_50_ provides the percentage of tumor volume with SAR above 50% of the maximum SAR in the breast [22]. The TC_50_ quantifies selective power deposition inside the tumor.

T_50_ and T_90_ were used in thermal simulation to evaluate the treatment effect [21]. T_50_ and T_90_ represent the lowest temperature reached at 50% and 90% of the tumor volume, respectively. The damaged healthy tissue rate [28,29] is expressed as

(8) $$\mathrm{Damaged}\;\mathrm{healthy}\;\mathrm{tissue}\;\mathrm{rate}\left(\%\right)=\left(\frac{V_{\mathrm{Damaged}\;\mathrm{healthy}\;\mathrm{tissue}}}{V_{\mathrm{Healthy}\;\mathrm{tissue}}}\right)\times 100\%$$

The damaged healthy tissue rate is used to observe the ability of the optimization algorithm to reduce hotspots in healthy tissue.

### 2.4. Optimization Methods of HTP

In this section, the optimization algorithms applied in HTP are introduced. The objective function of all optimization algorithms is HTQ, and the number of iterations and individuals is 200. Meanwhile, the range of amplitude is from 0 W to 1 W and the range of phase is from −180 degrees to 180 degrees. The power scaling factor is set as $v\in \left[2,100\right]$ [20]. The amplitudes ($A=\left\{a_{1},\ldots ,a_{n}\right\}$) optimized by optimization algorithms are transferred to the phased array applicator. The power of the phased array applicator was scaled uniformly according to *v*, which was gradually increased starting as 2 until the maximum steady-state temperature in the healthy tissue reached 44 °C [21].

#### 2.4.1. Particle Swarm Optimization (PSO)

PSO seeks the global optimal solution by tracking the optimal solution of the current search. PSO is used to solve practical problems because of its advantages such as simple implementation and fast convergence. When optimizing HTP by PSO, the amplitude $a_{n}$ and phase $\theta_{n}$ (*n* = 1, 2, …, *N*) of the *N* elements in the phased array form a particle. The objective function is HTQ, which is calculated by the SAR distribution simulated by EM. The position of the optimal fitness value experienced by the *t*-th particle is denoted as the particle best value *pbest*, and the optimal position experienced by the whole population is denoted as the global best value *gbest*. The *j* velocity of the particle *i* after *t*-th iteration is

(9) $$v_{ij}^{t}=w\cdot v_{ij}^{t-1}+c_{1}\cdot rand_{1}\cdot (pbest_{ij}^{t-1}-x_{ij}^{t-1})+c_{2}\cdot rand_{2}\cdot (gbest_{j}^{t-1}-x_{ij}^{t-1})$$

where $v_{ij}^{t-1}$ and $x_{ij}^{t-1}$ are the velocity and position of iteration (*t* − 1)-th, respectively. $c_{1}$ and $c_{2}$ are learning factors [30]. *w* is inertia weight and presents the proportion of velocity updates in the new iteration. The new position of the particle is updated as

(10) $$x_{ij}^{t}=x_{ij}^{t-1}+v_{ij}^{t}$$

In this paper, $c_{1}$, $c_{2}$, and w are set as 0.5, 1.5, and 0.9, respectively. The optimized operation is finished until the optimal target value or the maximum number of iterations is reached. Finally, the optimal solution in the population is derived.

#### 2.4.2. Genetic Algorithm (GA)

GA is a series of search algorithms inspired by natural evolutionary theory. In each iteration of GA, the individual is evaluated using the objective function. The parameters required for the best solution are generated for the next generation by selection, crossover, and mutation.

When optimizing HTP by GA, the combination of amplitude $a_{n}$ and phase $\theta_{n}$ of each antenna presents a chromosome. The corresponding complex phasor of phase and amplitude is expressed as [21]

(11) $$x_{n}=a_{n}\times e^{-i\varphi_{n}}$$

The individual is an *N*-dimensional vector composed of chromosomes, that is, the excitation of the phased array represents the individual. The population is composed of a number of chromosomes. In each generation, the objective function value of each chromosome is evaluated and the best one will be inherited by the next generation, replacing the current population with a new one generated by selection, crossover, and mutation [31]. The operation is repeated until the termination condition is satisfied, or the maximum number of iterations is reached. Meanwhile, the best result is selected from the current population. The crossover probability is set to 0.8.

#### 2.4.3. Differential Evolution (DE)

The HTP optimization algorithm in this paper is the differential evolution algorithm, which is a heuristic stochastic search algorithm based on population differences. DE was proposed by R. Store and K. Price for solving Chebyshev polynomials. Similar to other global optimization algorithms, the initial population of DE randomly generates individuals. The genetic process of the population is carried out through crossover, mutation. and selection to minimize the objective function. The relationship between hyperthermia parameters and DE parameters is similar to that of GA. The difference between the DE algorithm and the GA algorithm is the differential mutation process. The phase or amplitude of the phased array element presents the gene, the excitation (phase and amplitude) of each element is the chromosome, and one phased array configuration represents the individual. The DE optimization process is as follows:

1. Mutation

The *g-*th iteration produces a variant individual $Y_{i}$ of the target individual $X_{i}$ as follows

(12) $$Y_{i}(g)=X_{r0}(g)+F\times \left(X_{r1}(g)-X_{r2}(g)\right)$$

where $X_{r0}$, $X_{r1}$, and $X_{r2}$ are randomly selected in the population, and they are not equal to each other. *F* is the differential weight factor, which is set as 0.85.

2. Crossover

In the crossover operation, each individual and its generated offspring mutation vector are crossed. The crossover probability CR (set as 0.8) determines the proportion of chromosomes replicated from the mutant offspring to the new one.

(13) $$v_{i,j}=\left\{\begin{array}{l} h_{i,j},rand(0,1)\;\leq CR\\ x_{i,j},else \end{array}\right.$$

The crossover operation of DE is different from that of GA. The crossover of DE is for one dimension of the whole population, while the crossover of GA is for each individual in the population.

3. Selection

According to the value of the objective function, the best individual is selected from the target individual and the test individual as the next generation.

(14) $$X_{i}(g+1)=\left\{\begin{array}{l} V_{i}(g),HTQ\left(V_{i}(g)\right)<HTQ\left(X_{i}(g)\right)\\ X_{i}(g),else \end{array}\right.$$

The procedure is repeated until a certain objective value or the maximum number of iterations is satisfied. The best result is output from the population. The parameters of each optimization algorithm were obtained according to experience and several optimization simulations to provide a balance between the search range and the computation time.

The loss of energy during propagation results in the reduction of TR focusing accuracy [15]. Therefore, global optimization algorithms are used in HTP to improve the treatment effect. Therefore, DE is used in this paper to optimize HTP to avoid unwanted hotspots and better target tumors. By comparing with other HTP optimization methods (TR, PSO, and GA), the advancement of DE is verified.

## 3. Simulation Results of HTP Optimization Methods

This section presents the simulation results of TR, PSO, GA and DE. The performance of four HTP optimization methods is discussed in terms of SAR distribution, treatment indicators, temperature distribution, and temperature indicators.

### 3.1. EM Simulation Results Comparison

TR is only optimized for electromagnetic simulation in HFSS, which adopts adaptive grid generation technology. The simulation results are stable, so there is no standard deviation in TR data. To verify the stability of the algorithms, the DE, GA, and PSO algorithms were respectively run 50 times, and the mean and standard deviation of treatment indicators for three optimization algorithms were calculated through 50 independent simulations. The mean and standard deviation of treatment indicators of the general and heterogeneous breast models optimized by four HTP optimization methods are illustrated in Table 2.

Compared with the statistics of the other HTP optimization methods, the mean and standard deviation of HTQ, aPA, TC_50_, and coverage rate of the DE algorithm have substantial performance. The treatment indicators of global optimization algorithms are significantly better than TR technology. Among all models, the DE has the best treatment indicators because it has the minimized HTQ value and the maximum aPA and TC_50_ value. The mean HTQ of DE is 3.8% to 15.9% lower than GA, and the mean aPA of DE is 1.6% to 9.7% higher than GA. In particular, DE provides the minimum standard deviation for two breast models at two resonant frequencies. Compared with other optimization algorithms, the standard deviation of DE indicates that it has significant stability. The results illustrate that DE is better than the GA algorithm currently used in the 0.434 GHz microwave hyperthermia phased array applicator [20], and can locate the globally optimal values of all breast models at all frequencies more frequently and consistently. Hence, among the HTP optimization methods, the DE algorithm has optimal treatment accuracy and stable results.

**Figure 2:**
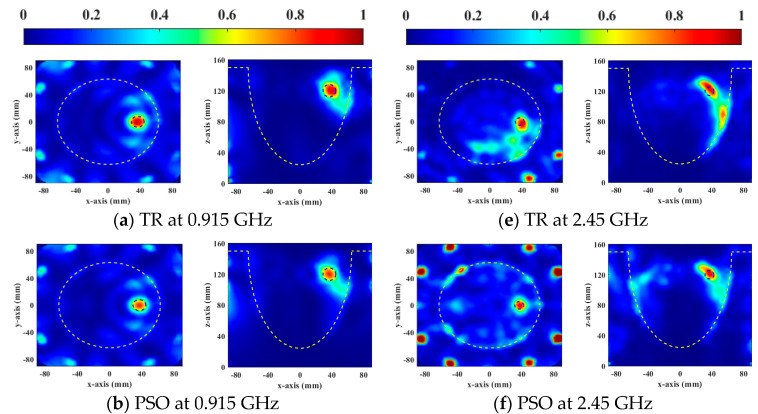

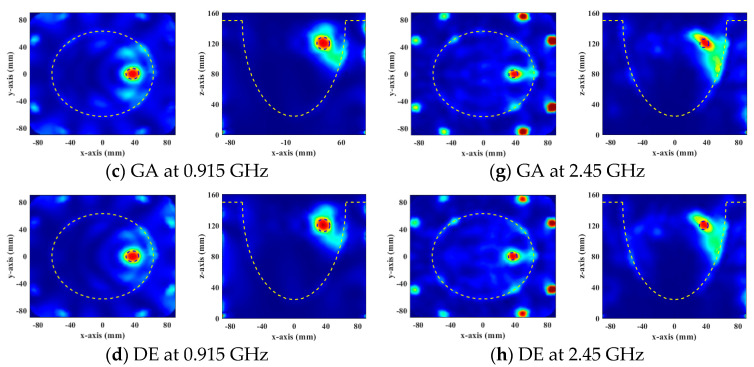
Normalized SAR distribution in axial (XY) and coronal (XZ) planes of a general breast model optimized using four FMHT optimization methods.

Figure 2 shows the SAR distributions of the optimal results of the four HTP optimization methods for focusing the general breast model at two resonant frequencies. The yellow and blue dotted lines represent the breast and the tumor boundaries, respectively. The cross-section is chosen based on the location of the tumor in the breast model. In Figure 2a,e, there are 2 mm and 4 mm focus shifts at 0.915 GHz and 2.45 GHz, respectively. All global optimization algorithms successfully focus energy on the target tumor. Compared with PSO, DE, and GA significantly increased the energy of the target tumor, so the HTQ of them were greater than PSO. The HTQ of DE was lower and aPA was higher, and the focusing range of DE in SAR distribution was more matched with the tumor, which proved that the energy difference between the tumor and the surrounding healthy tissue was large.

**Figure 3:**
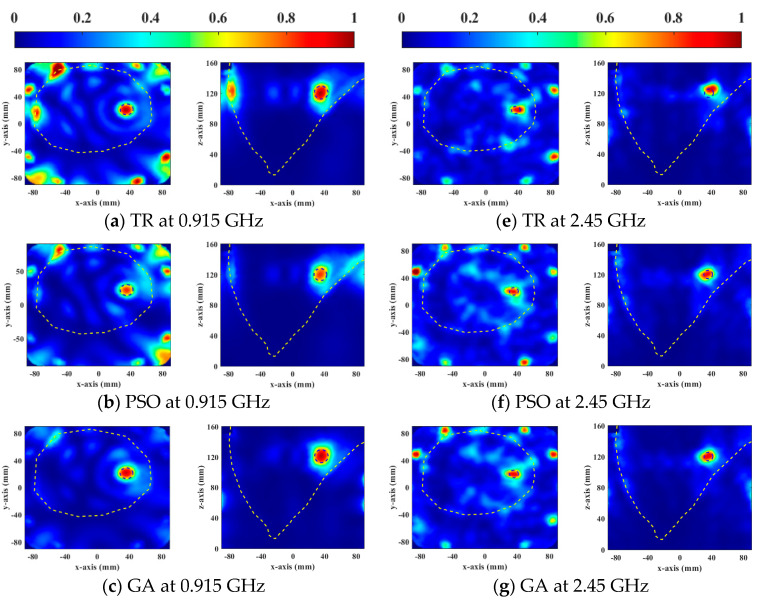

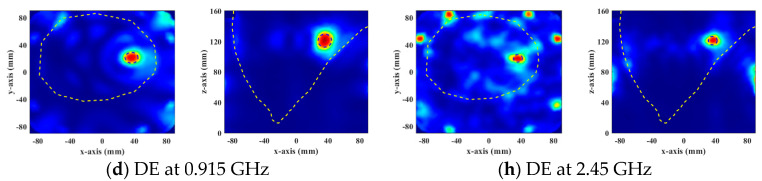
Normalized SAR distribution in axial (XY) and coronal (XZ) planes of a heterogeneous breast model optimized using four FMHT optimization methods.

The optimization ability of the DE algorithm is also better than other algorithms in the treatment of the heterogeneous breast model, as shown in Figure 3. Similarly, TR optimization results showed offsets and unexpected hotspots on the skin tissue. The results of global optimization algorithms are better than TR. The relatively low power in tumors with PSO results in higher HTQ and lower aPA. The SAR distribution of DE showed that there are no significant hotspots in healthy tissue, and the tumor targeting is accurate. Therefore, EM simulation results show that the DE algorithm has a better optimization effect than other HTP optimization methods for breast cancer.

### 3.2. Thermal Simulation Results Comparison

The mean and standard deviation of T_50_ and T_90_ of two breast models obtained by TR, PSO, GA, and DE as the HTP optimization methods are shown in Table 3. It can be observed that DE has the highest T_50_ and T_90_ compared to other HTP optimization methods. Due to the low power in the tumor of PSO optimization, the treatment temperature of PSO was the lowest. Compare with TR, the DE algorithm improves from 0.72 °C to 1.82 °C of T_50_ and 0.15 °C to 2.09 °C of T_90_. Therefore, the DE algorithm not only increases the effectiveness of focusing but also improves the temperature of tumor treatment. Table 4 shows the mean and standard deviation of damaged healthy tissue rate for two breast models using the four HTP optimization methods. In all cases, the average damaged healthy tissue rates of DE are the lowest among the four HTP optimization methods, and its standard deviations are the smallest. DE provided 16.13% and 9.75% fewer hotspots in 40–42 °C and 42–45 °C windows compared to GA, respectively. It should be noted that despite the low rate of healthy tissue damage at 0.915 GHz with PSO, the temperature of tumor treatment is also low.

**Figure 4:**
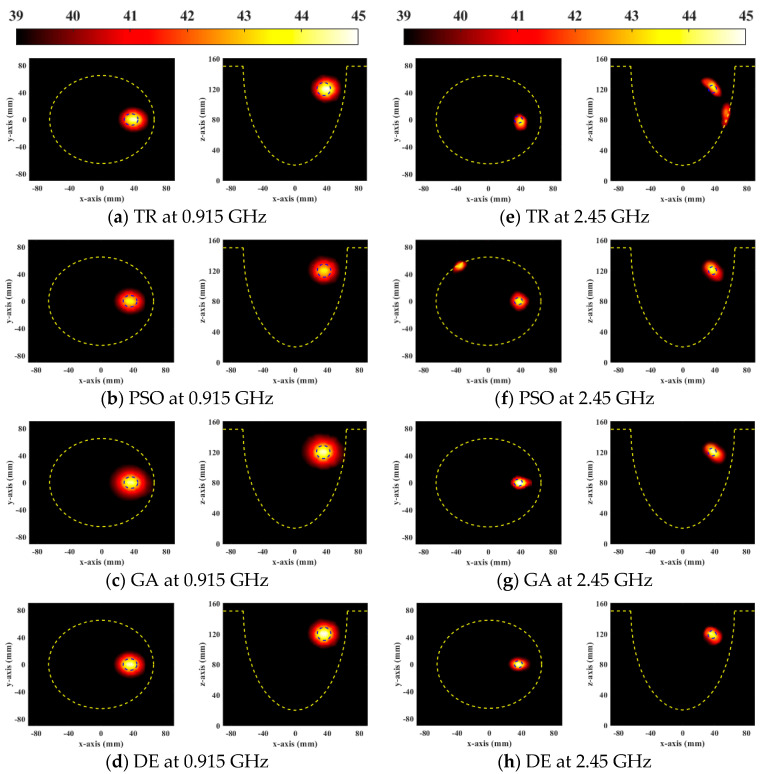
Steady-state temperature distribution in axial (XY) and coronal (XZ) planes of a general breast model treated by four FMHT optimization methods.

The temperature distribution more directly reflects the result of tumor treatment. Figure 4 shows steady-state temperature distributions of the general breast model optimized by four HTP optimization methods. As can be seen that the SAR distributions are similar to the focus of the temperature distributions. If the focus shifts and apparent excess hotspots exist in healthy tissues in EM simulation results, they also appear in thermal simulation results. TR optimization resulted in treatment location deviation, as shown in Figure 4a,e. When the PSO optimization algorithm was applied to HTP, the temperature in the tumor was the lowest among all the HTP optimization methods. It can be seen that the DE algorithm is superior to other optimization methods in temperature distribution, focused treatment range, and tumor treatment ability at two resonant frequencies.

**Figure 5:**
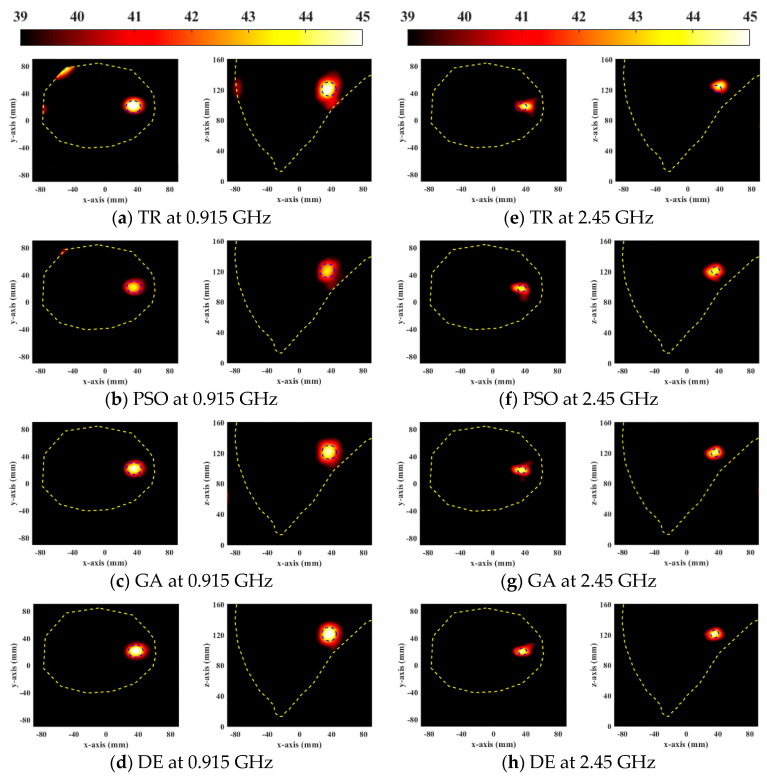
Steady-state temperature distribution in axial (XY) and coronal (XZ) planes of a heterogeneous breast model treated by four FMHT optimization methods.

Since the skin of the heterogeneous breast model is close to the antenna, the skin tissue near the antenna is at risk of being burned during microwave hyperthermia with TR, as shown in Figure 5a. However, the global optimization algorithm can reduce the energy of excess hot spots in healthy tissues, so the safety of healthy tissues can be ensured while treating breast cancer. As with the general breast model, the lowest tumor treatment temperature was obtained from the PSO algorithm. Treatment results showed that the DE algorithm was superior to other HTP optimization methods in the temperature distribution of the heterogeneous breast model. In summary, from the comparison of EM and thermal simulation results of the four HTP optimization methods, it can be seen that the DE algorithm greatly improves the therapeutic ability of microwave hyperthermia for breast cancer, provides excellent treatment temperature, and protects the health of normal breast tissue to the greatest extent.

### 3.3. Convergence Rate of Optimization Algorithms

Table 5 shows the convergence rate of PSO, GA, and DE to reach the PSO optimization result because TR had not been iteratively optimized. Theoretically, DE exhibits the fastest convergence and GA has the slowest convergence rate. Since GA and DE had a better-focusing effect than PSO in all simulation results, the optimal value of PSO was selected as the target value. Table 5 lists the number of iterations required for the three optimization algorithms to reach the same target value. Obviously, DE has the least number of iterations and GA has the slowest convergence rate in all cases. Hence, the DE algorithm has a better therapeutic effect, faster convergence rate, and better optimization stability than TR technology, PSO algorithm, and GA algorithm.

## 4. Influence of Objective Functions on DE Optimization

According to the above treatment results, the DE algorithm has better optimization ability than other optimization methods. The above optimization algorithm optimizes HTP based on HTQ as the objective function, in order to reduce HTQ and improve the energy ratio between the tumor and surrounding tissues. Since HTP optimization is based on SAR, all treatment indicators related to SAR can be used as the objective function of the optimization algorithm. Hence, HTQ*_obj_*, 1/aPA*_obj_*, and 1/TC_50*obj*_ were adopted as the objective functions of the DE algorithm to optimize the phased array excitation improves the tumor treatment effect. In order to select the optimal objective function of DE, EM, and thermal simulation were used to analyze the influence of different objective functions on the optimization effect of the DE algorithm.

Table 6 lists the treatment indicators of the heterogeneous breast model and the general breast model for the DE algorithm with three objective functions. The objective function HTQ*_obj_* has the lowest HTQ in all cases, indicating the maximum energy ratio between the tumor and surrounding tissue. In all cases, the objective function 1/aPA*_obj_* provided the highest aPA, with the aim of reducing power deposition and unnecessary hotspots in healthy tissue. Because the HTQ is low, it indicates that 1/aPA*_obj_* also reduces the energy of the tumor. The objective function 1/TC_50*obj*_ had the largest tumor coverage indicator TC_50_, but aPA and HTQ values were significantly worse than other objective functions.

**Figure 6:**
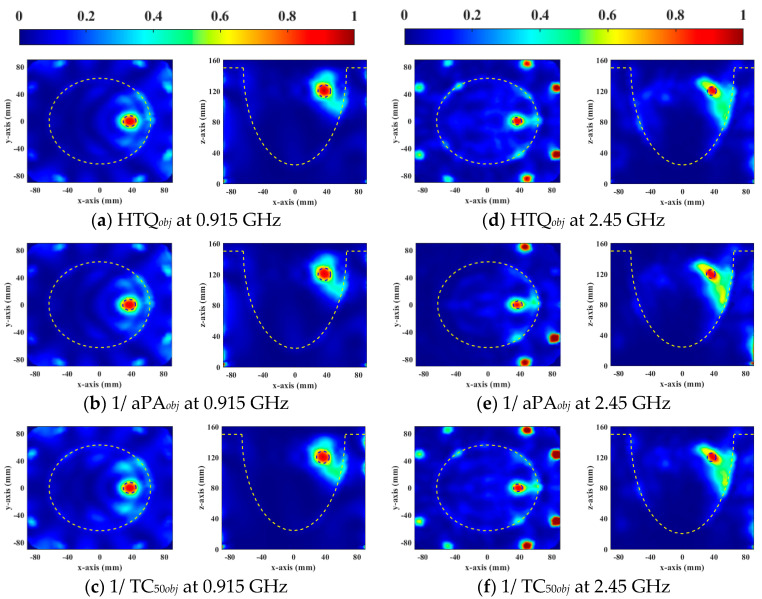
Normalized SAR distribution in axial (XY) and coronal (XZ) planes of a general breast model optimized by each objective function used in DE.

**Figure 7:**
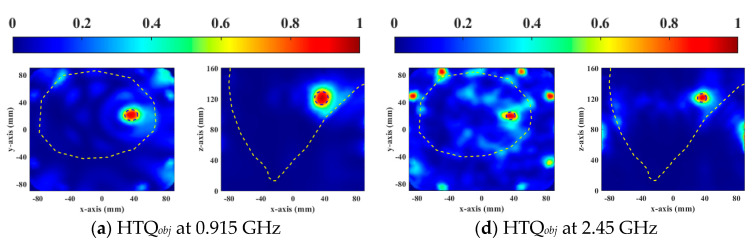

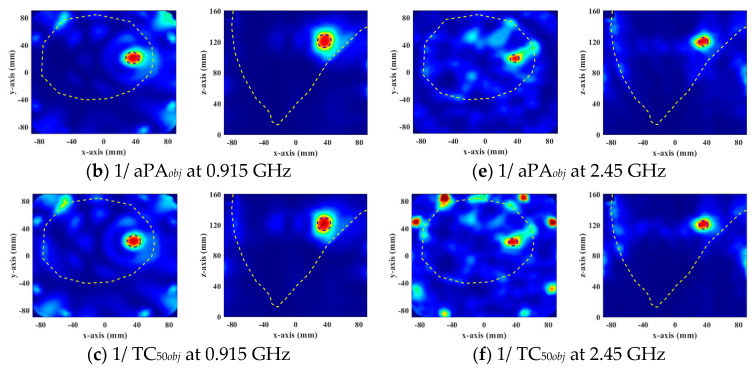
Normalized SAR distribution in axial (XY) and coronal (XZ) planes of a heterogeneous breast model optimized by each objective function used in DE.

Figure 6 and Figure 7 show the normalized SAR distributions of the general and the heterogeneous breast models for each objective function used in DE, respectively. When HTQ*_obj_* is the target function, there are no obvious redundant hot spots in the healthy tissue. Due to its low HTQ value, it indicates that the tissue energy ratio between the tumor and the surrounding tumor is large. When 1/aPA*_obj_* was the objective function used in DE, the hotspot in healthy tissue is the least. Because the HTQ value of 1/aPA*_obj_* is from 3.8% to 34% lower than HTQ*_obj_*, it indicates that the tissue energy ratio between the tumor and the surrounding tumor is low. When 1/TC_50*obj*_ was taken as the objective function of DE, the proportion of tumors in the treatment range was the largest and the highest unexpected hotspot was reached. Therefore, aPA of 1/TC_50*obj*_ is 1.8% to 7.4% lower than that of HTQ*_obj_* and the value of HTQ is 5.8% to 38.5% higher than that of HTQ*_obj_*. In EM simulation, the objective function HTQ*_obj_* provides the best HTP optimization using the DE algorithm for two breast models at two resonant frequencies.

The optimal treatment effect can be judged by the treatment temperature obtained by thermal simulation. Table 7 shows the T_50_ and T_90_ values of two breast models obtained by each objective function used in DE. It can be seen that HTQ*_obj_* and 1/aPA*_obj_* provided the highest and lowest treatment temperatures (T_50_ and T_90_) of all the objective functions, respectively. The T_50_ and T_90_ of DE with HTQ*_obj_* as the objective function are increased by 2% to 2.5% and 1.1% to 2.5% compared with other objective functions, respectively. In the optimization results of all objective functions of DE, the thermal damage to the surrounding healthy tissue was less than 5% in all cases (Table 8). In particular, HTQ*_obj_* objective function results showed the best protection for healthy tissue.

**Figure 8:**
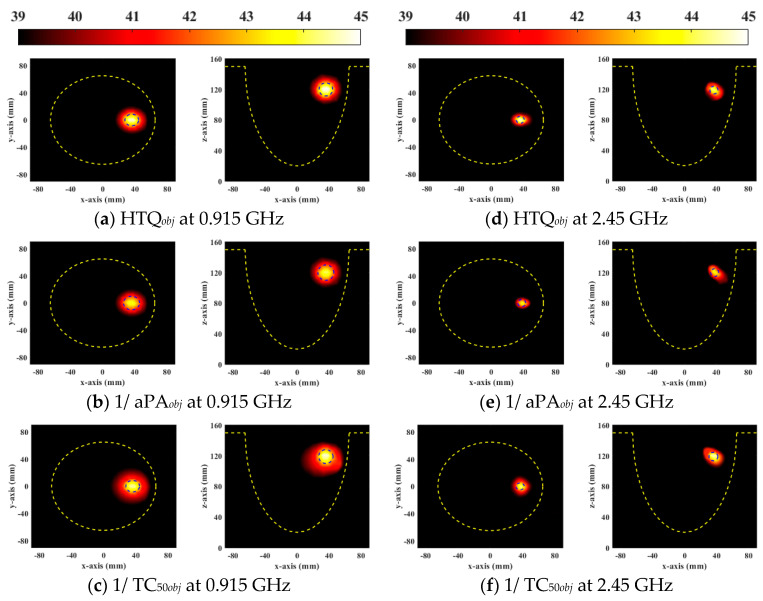
Steady-state temperature distribution in axial (XY) and coronal (XZ) planes of a general breast model optimized by each objective function used in DE.

**Figure 9:**
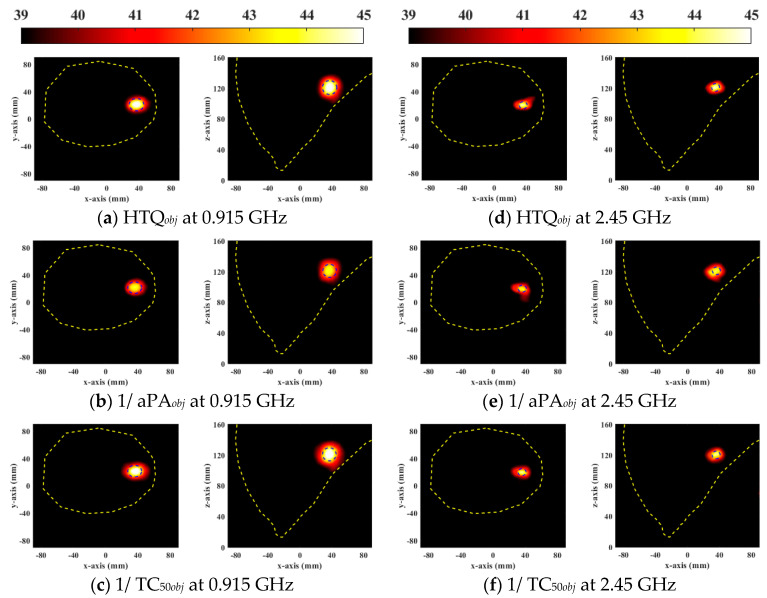
Steady-state temperature distribution in axial (XY) and coronal (XZ) planes of a heterogeneous breast model optimized by each objective function used in DE.

Steady-state temperature distribution can clearly observe the effect of breast cancer treatment. Figure 8 and Figure 9 show the steady-state temperature distributions of the general breast model and the heterogeneous breast model using different objective functions of the DE algorithm, respectively. It is observed that when HTQ*_obj_* is the objective function of the DE algorithm the temperature in tumors is highest and the treatment range is most similar to the tumor size for avoiding overtreatment. The temperature of the objective function 1/aPA*_obj_* is low relative to other objective functions caused by the lower energy deposited in the tumor. The objective function ignores the therapeutic power of the tumor by reducing hot spots in healthy tissue. However, when 1/TC_50*obj*_ was used as the target function, the treatment range completely included the tumor, but the surrounding tissue was damaged. This function ignores the health of surrounding tissue for increasing tumor treatment coverage. Therefore, the DE algorithm with objective function HTQ*_obj_* is the most effective to treat breast tumors, which can reduce the risk of damage to the healthy tissue of the patient while adequately treating tumors.

## 5. Discussion

The purpose of HTP is to accurately treat breast cancer with less damage to healthy tissue. A variety of optimization methods were used to optimize the phased array excitation to achieve the selective deposition of tumor energy. At present, global optimization algorithms are used to optimize phased array excitation under single-phase frequency to accurately treat tumors. According to the results of current articles on the PSO [9] and GA [11,20] algorithms used in microwave hyperthermia of breast cancer, the therapeutic results need to be further improved. Moreover, the influence of objective functions on the HTP optimization algorithm was ignored [21]. In addition, the damaged healthy tissue rate was not analyzed to verify the effectiveness of the DE algorithm in HTP [9].

In this work, we analyzed four microwave hyperthermia optimization methods to determine the best global optimization algorithm to generate HTP for tumor therapy and reduce hotspots in healthy tissues. The phased array applicator [15] operating at two resonant frequencies is used for treating 1 cm^3^ and 2 cm^3^ breast tumors in the upper outer quadrant of the general and heterogeneous breast models. Therefore, with HTQ as the objective function, the DE algorithm was used to optimize the powers and phases of the phased array applicator at 0.915 GHz and 2.45 GHz, and compared with TR technology, PSO, and GA algorithms. According to the comparison of the above data, the DE algorithm is superior to other optimization methods in hotspot reduction and limiting SAR focus targets.

The SAR-based treatment indicators and thermal results were calculated for evaluating the optimization ability of DE. DE decreased the HTQ value by 36.3% to 60% compared with TR in order to increase the power difference between tumor and healthy tissue. Compared with GA, which is the current best algorithm, DE reduced the HTQ value by 3.8% to 15.9%, the convergence rate of DE was significantly faster, and the damaged healthy tissue rate was the lowest. By verifying the EM and thermal results of three different objective functions used in DE for HTP, it can be concluded that HTQ*_obj_* is the most suitable objective function for DE.

The accuracy of the dual-resonant phased array applicator in the treatment of tumors of different sizes had been reflected in our previous work [15]. In this paper, we analyzed the optimization effect of the DE algorithm in HTP for breast cancer. However, the traditional first-order Arrhenius thermal damage equation [28], the modified Arrhenius equation for living tissues [32], the temperature-dependent time-delay equation [29], and other methods are used to quantify the thermal damage of healthy tissue and tumor. Since the uncertainty of thermal characteristics will affect the absolute temperature distribution [20], this study only discusses the relative differences of thermal indicators for the optimization algorithms commonly used in hyperthermia literature. Considering these factors, it is expected that the thermal model established can reliably predict the relative effects of different algorithms on thermal distribution. It is important to note that the applicability of this study to other phased array applicators requires rigorous validation of the respective phased array applicators and breast models. The HTP results depend on the basic radiator, phased array configuration, and treatment site.

## 6. Conclusions

In this paper, through the comparison of different HTP optimization methods, it is determined that the DE algorithm has the best optimization effect on breast cancer treatment, which further improves the treatment effect of dual resonance phased array applicator on 1 cm^3^ and 2 cm^3^ tumors. In particular, compared with TR technology, the DE algorithm significantly improves the treatment results of microwave hyperthermia for breast cancer. The treatment results of DE were consistently superior to TR, GA, and PSO, focusing most of the energy on the tumor and reducing hotspots in healthy tissues. By analyzing the influence of the SAR-based objective function on DE optimization, it is determined that the objective function HTQ can improve the therapeutic effect more than other objective functions used in DE. The DE algorithm with an objective function of HTQ has a good optimization effect in microwave hyperthermia of breast cancer, which can focus the most energy on the tumor while reducing hotspots in the surrounding healthy tissue.

In the future, we plan to focus on the development and application of the multi-objective differential evolution optimization algorithm to treat more real breast models and verify the optimization ability of DE for different phased array applicators.

## Figures and Tables

**Table 1:** Dielectric and thermal properties of breast tissue at 0.915 GHz and 2.45 GHz.

Freq. (GHz)		Breast Tissue	Fat	Skin	Chest	Fibro Glandular	Tumor
**Dielectric Properties**	0.915 [8]	Conductivity (S/m)	0.2	1.0	2.0	0.6	0.6
		Dielectric constant	15	40	65	55	55
	2.45 [9]	Conductivity (S/m)	0.15	1.0	2.0	0.5	0.5
		Dielectric constant	15	40	65	45	45
**Thermal** [11] **Properties**		Density (kg/m^3^)	1069	1085	1040	1050	1050
		Specific heat capacity (J/kg K)	2348	3391	3421	2960	2960
		Thermal conductivity (W/m K)	0.21	0.37	0.49	0.33	0.33
		Blood perfusion rate (1/s)	0.001	0.002	0.003	0.003	0.003
		Metabolic heat generation (W/m^3^)	350	1620	1046	690	690

**Table 2:** Mean and standard deviation of treatment indicators of two breast models optimized using four HTP optimization methods.

Breast Type	Freq. (GHz)	Treatment Indicator	TR	PSO	GA	DE
General	0.915	HTQ	0.79	0.72 ± 0.0741	0.52 ± 0.0098	0.50 ± 0.0051
		aPA	15.60	16.85 ± 1.36	19.66 ± 0.46	20.24 ± 0.15
		TC_50_	79	89 ± 2.60	98 ± 0.64	98 ± 0.35
	2.45	HTQ	1.36	0.81 ± 0.0503	0.69 ± 0.0128	0.58 ± 0.0062
		aPA	8.98	13.11 ± 1.12	16.28 ± 0.26	17.86 ± 0.06
		TC_50_	70	90 ± 1.89	96 ± 0.35	98 ± 0.26
Heterogenous	0.915	HTQ	1.26	0.81 ± 0.0126	0.53 ± 0.0047	0.50 ± 0.0008
		aPA	12.75	15.62 ± 1.16	20.36 ± 0.02	21.17 ± 0.05
		TC_50_	90	80 ± 3.15	99 ± 0.43	99 ± 0.24
	2.45	HTQ	1.96	0.69 ± 0.0776	0.55 ± 0.0215	0.51 ± 0.0154
		aPA	9.06	15.47 ± 0.94	17.95 ± 0.60	18.23 ± 0.37
		TC_50_	40	85 ± 2.46	96 ± 0.69	96 ± 0.44

**Table 3:** Mean and standard deviation of T_50_ and T_90_ values for two breast models using HTP optimization methods.

Breast Type	Freq. (GHz)	T (°C)	TR	PSO	GA	DE
General	0.915	T_50_	42.96	41.56 ± 2.37	43.16 ± 0.66	43.68 ± 0.03
		T_90_	41.75	40.39 ± 2.94	41.94 ± 0.97	42.46 ± 0.39
	2.45	T_50_	40.99	41.85 ± 1.35	42.03 ± 0.58	42.07 ± 0.32
		T_90_	39.77	40.59 ± 1.81	40.82 ± 0.69	41.86 ± 0.79
Heterogeneous	0.915	T_50_	42.98	41.24 ± 1.26	42.89 ± 0.47	43.16 ± 0.42
		T_90_	41.80	40.02 ± 1.96	41.70 ± 0.92	41.95 ± 0.85
	2.45	T_50_	41.06	41.72 ± 1.82	42.32 ± 0.71	42.61 ± 0.54
		T_90_	39.89	40.50 ± 2.46	41.11 ± 0.96	41.46 ± 0.65

**Table 4:** Mean and standard deviation of damaged healthy tissue rate for two breast models using HTP optimization methods.

Breast Type	Freq. (GHz)	T (%)	TR	PSO	GA	DE
General	0.915	40–42 °C	1.78	1.78 ± 2.23	3.31 ± 0.87	2.05 ± 0.65
		42–44 °C	1.32	0.28 ± 0.58	0.96 ± 0.38	0.82 ± 0.31
	2.45	40–42 °C	1.59	1.8 ± 1.63	1.44 ± 0.79	1.23 ± 0.23
		42–44 °C	1.11	0.99 ± 0.56	0.78 ± 0.26	0.36 ± 0.07
Heterogeneous	0.915	40–42 °C	4.02	1.60 ± 2.05	1.85 ± 0.85	1.78 ± 0.51
		42–44 °C	2.24	0.27 ± 0.76	0.43 ± 0.32	0.68 ± 0.19
	2.45	40–42 °C	1.05	1.29 ± 1.20	0.91 ± 0.64	0.83 ± 0.33
		42–44 °C	0.72	0.36 ± 0.51	0.53 ± 0.22	0.22 ± 0.13

**Table 5:** Iteration number of three algorithms to reach PSO’s optimum solution.

Breast Type	Frequency (GHz)	PSO	GA	DE
General	0.915	91	106	63
	2.45	113	134	80
Heterogeneous	0.915	85	115	76
	2.45	78	108	52

**Table 6:** Mean and standard deviation of treatment indicators of two breast models for each objective function used in DE.

Breast Type	Freq. (GHz)	Treatment Indicator	HTQ*_obj_*	1/aPA*_obj_*	1/TC_50*obj*_
General	0.915	HTQ	0.52	0.54	0.55
		aPA	21.03	21.18	20.65
		TC_50_	98	98	99
	2.45	HTQ	0.65	0.87	0.90
		aPA	16.89	17.22	16.46
		TC_50_	97	97	98
Heterogeneous	0.915	HTQ	0.50	0.53	0.55
		aPA	21.19	21.97	19.63
		TC_50_	100	100	100
	2.45	HTQ	0.50	0.67	0.68
		aPA	18.56	18.89	18.13
		TC_50_	96	96	97

**Table 7:** The values of T_50_ and T_90_ of each objective function used in DE optimized the dual-resonant phased array excitations.

Breast Type	Frequency (GHz)	T (°C)	HTQ*_obj_*	1/aPA*_obj_*	1/TC_50*obj*_
General	0.915	T_50_	43.70	42.52	42.98
		T_90_	42.52	41.34	41.73
	2.45	T_50_	42.42	41.03	41.67
		T_90_	41.26	40.46	40.83
Homogeneous	0.915	T_50_	43.62	42.29	43.15
		T_90_	42.86	41.71	41.90
	2.45	T_50_	43.21	42.03	42.66
		T_90_	42.00	41.23	41.85

**Table 8:** Mean and standard deviation of damaged healthy tissue rate for two breast models using DE.

Breast Type	Frequency (GHz)	T (%)	HTQ*_obj_*	1/aPA*_obj_*	1/TC_50*obj*_
General	0.915	40–42 °C	1.72	1.52	3.40
		42–44 °C	0.60	0.07	1.01
	2.45	40–42 °C	1.19	0.70	1.31
		42–44 °C	0.33	0.13	0.53
Homogeneous	0.915	40–42 °C	1.62	1.28	2.13
		42–44 °C	0.66	0.01	0.69
	2.45	40–42 °C	0.75	0.90	0.82
		42–44 °C	0.20	0.36	0.22

## Data Availability

Not applicable.
